# Race and Ethnicity and Primary Language in Emergency Department Triage

**DOI:** 10.1001/jamanetworkopen.2023.37557

**Published:** 2023-10-12

**Authors:** Joshua W. Joseph, Maura Kennedy, Alden M. Landry, Regan H. Marsh, Da’Marcus E. Baymon, Dana E. Im, Paul C. Chen, Margaret E. Samuels-Kalow, Lauren M. Nentwich, Noémie Elhadad, León D. Sánchez

**Affiliations:** 1 Department of Emergency Medicine, Brigham and Women’s Hospital, Boston, Massachusetts; 2 Harvard Medical School, Boston, Massachusetts; 3 Department of Emergency Medicine, Massachusetts General Hospital, Boston; 4 Department of Emergency Medicine, Beth Israel Deaconess Medical Center, Boston; 5 Departments of Biomedical Informatics and Computer Science, Columbia University, New York, New York

## Abstract

**Importance:**

Emergency department (ED) triage substantially affects how long patients wait for care but triage scoring relies on few objective criteria. Prior studies suggest that Black and Hispanic patients receive unequal triage scores, paralleled by disparities in the depth of physician evaluations.

**Objectives:**

To examine whether racial disparities in triage scores and physician evaluations are present across a multicenter network of academic and community hospitals and evaluate whether patients who do not speak English face similar disparities.

**Design, Setting, and Participants:**

This was a cross-sectional, multicenter study examining adults presenting between February 28, 2019, and January 1, 2023, across the Mass General Brigham Integrated Health Care System, encompassing 7 EDs: 2 urban academic hospitals and 5 community hospitals. Analysis included all patients presenting with 1 of 5 common chief symptoms.

**Exposures:**

Emergency department nurse-led triage and physician evaluation.

**Main Outcomes and Measures:**

Average Triage Emergency Severity Index [ESI] score and average visit work relative value units [wRVUs] were compared across symptoms and between individual minority racial and ethnic groups and White patients.

**Results:**

There were 249 829 visits (149 861 female [60%], American Indian or Alaska Native 0.2%, Asian 3.3%, Black 11.8%, Hispanic 18.8%, Native Hawaiian or Other Pacific Islander <0.1%, White 60.8%, and patients identifying as Other race or ethnicity 5.1%). Median age was 48 (IQR, 29-66) years. White patients had more acute ESI scores than Hispanic or Other patients across all symptoms (eg, chest pain: Hispanic, 2.68 [95% CI, 2.67-2.69]; White, 2.55 [95% CI, 2.55-2.56]; Other, 2.66 [95% CI, 2.64-2.68]; *P* < .001) and Black patients across most symptoms (nausea/vomiting: Black, 2.97 [95% CI, 2.96-2.99]; White: 2.90 [95% CI, 2.89-2.91]; *P* < .001). These differences were reversed for wRVUs (chest pain: Black, 4.32 [95% CI, 4.25-4.39]; Hispanic, 4.13 [95% CI, 4.08-4.18]; White 3.55 [95% CI, 3.52-3.58]; Other 3.96 [95% CI, 3.84-4.08]; *P* < .001). Similar patterns were seen for patients whose primary language was not English.

**Conclusions and Relevance:**

In this cross-sectional study, patients who identified as Black, Hispanic, and Other race and ethnicity were assigned less acute ESI scores than their White peers despite having received more involved physician workups, suggesting some degree of mistriage. Clinical decision support systems might reduce these disparities but would require careful calibration to avoid replicating bias.

## Key Points

**Question:**

Are there significant differences in triage Emergency Severity Index scores and the intensity of physician evaluations (as measured by work relative value units) provided to Black patients, Hispanic patients, and those identifying as Other racial and ethnic background compared with White patients?

**Findings:**

In this cross-sectional study of 249 829 visits to 7 academic and community hospital emergency departments, Patients identifying as Black, Hispanic, and Other tended to have less acute triage scores than their White peers. Despite having the same chief symptoms, Patients identifying as Black, Hispanic, and Other received more intense physician evaluations.

**Meaning:**

These findings suggest the need to address systemic disparities in the emergency department triage process.

## Teaser

This cross-sectional study examines emergency department triage and resultant workup by physicians among patients of differing races and ethnicities.

## Introduction

Emergency department (ED) triage is a difficult yet critical aspect of ED care, requiring years of experience and training to perform accurately. Relatively few objective criteria exist within the standard Emergency Severity Index (ESI) triage algorithm used in most EDs throughout the US, leaving triage nurses considerable latitude in determining a patient’s ESI score, and by extension, the priority with which they are evaluated by physicians.^1,2^ In light of sustained rates of high ED crowding and boarding, when patients often may face delays of multiple hours to see a clinician, triage can play an outsize role in determining the timeliness of patient care.

Several studies have documented major disparities in the assignment of ESI triage scores to ED patients according to their race and ethnicity.^3,4,5,6^ Specifically, Black and Hispanic patients tend to be assigned lower-acuity triage scores than were White patients, even with identical chief symptoms, such as abdominal pain.^3,4,5^ A recent study has also reported that Black patients wait longer to be brought back into the ED even when assigned the same triage scores as White patients.^6^

These disparities are paralleled by a tendency among physicians to provide patients of minority race and ethnicity with fewer diagnostic and therapeutic interventions than their White peers. In particular, studies have observed that physicians tend to order fewer computed tomography scans and other advanced imaging studies for Black and Hispanic patients.^7,8,9^ Other studies have also reported that physicians order fewer analgesic medications for both Black and Hispanic patients.^10,11^

In this study, we sought to further examine the findings of a recent study reporting persistent racial and ethnic disparities in the triage ESI scores and the intensity of physician evaluations for patients with common chief symptoms at the ED of an academic hospital,^12^ examining whether these findings may be replicated across a network comprising multiple academic and community hospital settings.

## Methods

### Study Design

This cross-sectional study examined visits of adults presenting with 1 of a group of 5 common chief symptoms used in a previous study of emergency triage.^12^ The study was granted an exemption from the use of informed consent by the Mass General Brigham Institutional Review Board owing to the use of deidentified, aggregated data. This study followed the Strengthening the Reporting of Observational Studies in Epidemiology (STROBE) reporting guideline.

### Study Setting and Population

All unique adult patient visits between February 28, 2019, and January 1, 2023, to EDs within the Mass General Brigham Integrated Health Care System were screened for inclusion in the study. The network evaluated in our study included 7 EDs: 2 large urban academic hospitals and 5 community hospitals located in urban and suburban settings throughout the Northeastern US. Average yearly visit volumes across the network range from 15 000 to greater than 117 760 encounters per year. The ESI is used for triage in all EDs included within the study and performed by a trained ED nurse. At the academic sites, attending physicians and midlevel clinicians may perform posttriage screening evaluations during portions of the day, but they do not assign ESI scores.

All patients presenting with standardized chief symptoms of chest pain, shortness of breath, abdominal pain, fall, and nausea/vomiting, or a direct synonym of one of these symptoms (ie, chest discomfort, dyspnea) were included. These are common reasons for visiting the ED and can be associated with serious disorders. In the case of both chest pain and shortness of breath, they are cited in ESI training materials as examples of symptoms that are likely to merit acute triage designations.^2^ The ESI training materials provide individual nurses considerable latitude to use their clinical judgment, with the presence of specific abnormal vital sign thresholds as triggers to consider ranking patients at ESI level 2.

### Variables

All patients are assigned an ESI score at the time of initial ED triage. The ESI scores are in a 5-point range, from level 1 (most urgent) to level 5 (least urgent). The ESI score indicates the urgency with which a patient should be evaluated by the physician, but also factors in the resources a patient is expected to require.

In the ED, work relative value units (wRVUs) serve as a surrogate measure of the intensity of a patient’s diagnostic workup and any procedures or therapeutic interventions that are provided. Physician evaluations and procedures are assigned specific wRVU values based on their perceived effort and complexity. While the number of wRVUs is influenced by the thoroughness of physician documentation, the use of diagnostic interventions (eg, computed tomography scans) and therapeutic interventions (eg, intravenous narcotics) will automatically increase the evaluation and management level associated with the visit, which provides a substantial portion of the total wRVUs associated with a patient’s visit.^12^

Demographic variables collected at triage included age, gender, whether the patient had an in-network primary care physician, primary language, and self-reported race and ethnicity (American Indian or Alaska Native, Asian, Black or African American, Hispanic, Native Hawaiian or other Pacific Islander, non-Hispanic White [hereafter, White], and Other race or ethnicity). To align our analysis with previous studies, the Hispanic category encompassed patients who indicated both White race and Hispanic ethnicity, whereas non-White Hispanic patients were included within their primary self-identified racial category. Patients may self-identify their race as Other or 2 or more without needing to further specify when they do not feel that the provided categories adequately describe their identity. The 2 or more category defaults to Other in our database and does not specify the racial and ethnic backgrounds. We included these patients as well as those who declined to self-identify within the Other category for the purpose of analysis.

### Statistical Analysis

We evaluated differences in ESI scores and total visit wRVUs between self-identified racial and ethnic groups compared with White patients using stratified independent *t* tests, with mean values and 95% CIs as descriptive statistics. An overall significance level of *P* < .001, determined with a 2-sided independent *t* test, was used to provide a strict Bonferroni correction for 6 sets of comparisons across racial and ethnic groups for 5 chief symptoms. To capture differences in physician evaluation independent of triage ESI scores (levels 1 and 2), we conducted a secondary analysis for patients assigned ESI level 3 only. In light of the influence that older age has been shown to have on morbidity, mortality, and increased diagnostic intensity, we also evaluated wRVUs stratifying for age 65 years or older and age 50 years or older.^12,13^ An additional analysis evaluated for the primary language and whether the patient had an in-network primary care physician. All analyses were performed in Python, version 3.9 (Python Software Foundation) using the SciPy library.^14^

## Results

A total of 249 829 visits for patients greater than or equal to age 18 years (149 861 women [60%]; 99 968 men [40%]); median age, 48 years [IQR, 29-66 years]) were included in the study. Patient characteristics for the full group and divided by racial and ethnic groups are described in Table 1. A total of 0.2% patients were American Indian or Alaska Native, 3.3% were Asian, 11.8% were Black, 18.8% were Hispanic, less than 0.1% were Native Hawaiian or Other Pacific Islander, 60.8% were White, and 5.1% indicated Other race or ethnicity. White patients were significantly older, more likely to have English as their primary language, and more likely to have an in-network primary care physician than patients in any other racial or ethnic group.

**Table 1.** Characteristics of the Study Population

Characteristic	Patients, No. (%)									
	All (N = 249 829)	Gender		Race and ethnicity						
		Male (n = 99 968 [40%])	Female (n = 149 861 [60%])	American Indian or Alaska Native (n = 434 [0.2%])	Asian (n = 8287 [3.3%])	Black (n = 29 404 [11.8%])	Hispanic (n = 46 968 [18.8%])	Native Hawaiian or Other Pacific Islander (n = 132 [<0.1%])	Other (n = 12 623 [5.1%])^a^	White (n = 151 981 [60.8%])
Age, median, (IQR), y	48 (29-66)	48 (29-66)	50 (30-65)	39 (25-59)	39 (25-59)	41 (28-57)	35 (24-51)	36 (31-50)	38 (23-57)	56 (35-71)
Female	149 861 (60)	0	149 861 (100)	270 (62.2)	5096 (61.5)	18 588 (63.2)	29 859 (63.6)	96 (72.7)	7428 (58.8)	88 524 (58.2)
Male	99 968 (40)	99 968 (40)	0	164 (37.8)	3191 (38.5)	10 816 (36.8)	17 109 (36.4)	36 (27.3)	5195 (41.2)	63 457 (39.2)
English as primary language	213 917 (85.6)	86 772 (86.8)	127 145 (84.8)	422 (97.2)	6319 (76.3)	26 283 (89.4)	24 206 (51.5)	126 (95.5)	9057 (71.8)	147 482 (97.0)
ESI score, median, (IQR)^b^	3 (2-3)	3 (2-3)	3 (3-3)	3 (3-3)	3 (2-3)	3 (3-3)	3 (3-3)	3 (2-3)	3 (3-3)	3 (2-3)
Abdominal pain	86 147 (34.5)	30 242 (30.3)	55 905 (37.3)	141 (32.5)	3286 (39.7)	10 879 (37.0)	20 840 (44.4)	42 (31.8)	4716 (37.4)	46 243 (30.4)
Chest pain	65309 (26.1)	29 961 (30.0)	35 348 (23.6)	110 (25.3)	2196 (26.5)	8718 (29.7)	11 841 (25.2)	49 (37.1)	3169 (25.1)	39 226 (25.8)
Shortness of breath	33 797 (13.5)	14 759 (14.8)	19 038 (12.7)	78 (18.0)	970 (11.7)	4137 (14.1)	5505 (11.7)	18 (13.6)	1685 (13.3)	21 404 (14.1)
Nausea or vomiting	15 497 (6.2)	5215 (5.2)	10 282 (6.9)	29 (6.7)	485 (5.9)	2218 (7.5)	3341 (7.1)	11 (8.3)	821 (6.5)	8592 (5.7)
Fall	49 055 (19.6)	19 782 (19.8)	29 273 (19.5)	76 (17.5)	1349 (16.3)	3449 (11.7)	5436 (11.6)	12 (9.1)	2231 (17.7)	36 502 (24.0)
ESI 1	685 (0.3)	374 (0.4)	311 (0.2)	0	24 (0.3)	62 (0.2)	66 (0.1)	0	45 (0.4)	488 (0.3)
ESI 2	61 810 (24.7)	27 848 (27.9)	33 962 (22.7)	40 (30.2)	2079 (25.1)	6797 (23.1)	8733 (18.6)	40 (30.2)	2657 (21.1)	41 401 (27.2)
ESI 3	171 404 (68.6)	65 227 (65.2)	106 177 (70.9)	88 (66.4)	5620 (67.8)	20 733 (70.5)	34 417 (73.3)	88 (66.4)	8905 (70.5)	101 341 (66.7)
ESI 4	15 647 (6.3)	6383 (6.4)	9264 (6.2)	5 (3.4)	546 (6.6)	1771 (6.0)	3661 (7.8)	5 (3.4)	992 (7.9)	8640 (5.7)
ESI 5	283 (0.1)	136 (0.1)	147 (0.1)	0	17 (0.2)	41 (0.1)	91 (0.2)	0	24 (0.2)	110 (0.1)

Abbreviation: ESI, Emergency Severity Index.

^a^ Other race and ethnicity category is patient-defined. It was available for any patient who did not feel that the predefined categories were appropriate.

^b^ The ESI scores are in a 5-point range, from level 1 (most urgent) to level 5 (least urgent). The ESI score indicates the urgency with which a patient should be seen but also factors in the resources a patient is expected to require.

The 5 common chief symptoms—abdominal pain (34.5%), chest pain (26.1%), falls (19.6%), shortness of breath (13.5%), nausea or vomiting (6.2%)—were predominantly assigned ESI scores of 2 and 3. Both chest pain and shortness of breath were typically assigned more acute ESI scores, in keeping with the recommendations of the ESI triage algorithm. Approximately 6308 visits (2.5%) represent repeated presentations by patients with the same chief symptoms during the 4-year study period.

For all chief symptoms, White patients were assigned more acute or equal triage ESI scores compared with patients of any other racial or ethnic group. White patients were assigned more acute triage ESI scores than patients who identified as Hispanic or Other for all 5 conditions (eg, chest pain: Hispanic, 2.68 [95% CI, 2.67-2.69]; White, 2.55 [95% CI, 2.55-2.56]; Other, 2.66 [95% CI, 2.64-2.68]; *P* < .001) (Table 2). White patients were assigned more acute scores than Black patients for chest pain, abdominal pain, falls, and nausea/vomiting (Black, 2.97 [95% CI, 2.96-2.99]; White: 2.9 [95% CI, 2.89-2.91]; *P* < .001); there was no statistically significant difference for shortness of breath. Statistically significant differences in ESI scores were seen between White and Asian patients only for falls (mean, Asian, 3.01 [95% CI, 2.97-3.06], White, 2.91 [95% CI, 2.90-2.92]; *P* < .001). No significant differences were seen between White and American Indian or Alaska Native patients, or Native Hawaiian or Other Pacific Islander patients, although assessment of these groups was limited by sample sizes.

**Table 2.** Emergency Severity Index and Visit Work Relative Value Unit Differences Between Racial and Ethnic Groups Compared With White Patients

Chief symptom	American Indian or Alaska Native	Asian	Black	Hispanic	Native Hawaiian or Other Pacific Islander	Other^a^	White
**Emergency Severity Index score, mean (95% CI)**							
Abdominal pain	2.92 (2.86-2.98)	2.9 (2.88-2.91)	2.92 (2.92-2.93)	2.96 (2.96-2.97)	2.92 (2.86-2.98)	2.93 (2.92-2.94)	2.88 (2.88-2.89)
*P* value	.33	.12	<.001	<.001	.44	<.001	NA
Chest pain	2.62 (2.52-2.73)	2.59 (2.56-2.61)	2.62 (2.61-2.63)	2.68 (2.67-2.69)	2.62 (2.52-2.73)	2.66 (2.64-2.68)	2.55 (2.55-2.56)
*P* value	.19	.01	<.001	<.001	.61	<.001	NA
Shortness of breath	2.72 (2.59-2.85)	2.74 (2.7-2.78)	2.7 (2.69-2.72)	2.79 (2.78-2.81)	2.72 (2.59-2.85)	2.78 (2.75-2.81)	2.7 (2.69-2.71)
*P* value	.77	.05	.56	<.001	.71	<.001	NA
Nausea or vomiting	3.04 (2.86-3.22)	2.91 (2.87-2.96)	2.97 (2.96-2.99)	3.02 (3.01-3.04)	2.82 (2.58-3.06)	2.99 (2.95-3.02)	2.90 (2.89-2.91)
*P* value	.09	.50	<.001	<.001	.53	<.001	NA
Fall	3.03 (2.85-3.21)	3.01 (2.97-3.06)	3.11 (3.08-3.13)	3.14 (3.12-3.17)	3.22 (2.79-3.66)	3.04 (3.01-3.08)	2.91 (2.90-2.92)
*P* value	.15	<.001	<.001	<.001	.16	<.001	NA
**Work relative value units**							
Abdominal pain	4.08 (3.46-4.71)	4.02 (3.92-4.12)	4.51 (4.46-4.57)	4.26 (4.22-4.3)	4.73 (3.76-5.7)	4.06 (3.97-4.15)	3.98 (3.95-4.01)
*P* value	.68	.45	<.001	<.001	.11	.08	NA
Chest pain	3.41 (2.83-3.99)	3.75 (3.61-3.89)	4.32 (4.25-4.39)	4.13 (4.08-4.18)	3.87 (3.14-4.61)	3.96 (3.84-4.08)	3.55 (3.52-3.58)
*P* value	.66	.006	<.001	<.001	.50	<.001	NA
Shortness of breath	3.16 (2.60-3.72)	3.72 (3.49-3.95)	4.13 (4.04-4.22)	3.87 (3.79-3.95)	4.85 (2.96-6.75)	3.54 (3.38-3.7)	3.54 (3.5-3.59)
*P* value	.31	.12	<.001	<.001	.10	.94	NA
Nausea or vomiting	3.37 (2.49-4.26)	3.58 (3.31-3.84)	3.91 (3.79-4.02)	3.54 (3.46-3.62)	3.36 (2.59-4.13)	3.39 (3.19-3.58)	3.6 (3.53-3.66)
*P* value	.70	.87	<.001	.32	.80	.06	NA
Fall	3.37 (2.66-4.08)	4.5 (4.21-4.79)	4.6 (4.47-4.74)	4.44 (4.32-4.55)	4.26 (1.71-6.82)	4.43 (4.24-4.63)	4.53 (4.49-4.58)
*P* value	.02	.79	.37	.13	.84	.31	NA

Abbreviation: NA, not applicable.

^a^ Other race and ethnicity category is patient defined. It was available for any patient who did not feel that the predefined categories were appropriate.

When stratifying for patients older than 65 years, these differences remained significant. White patients were assigned more acute ESI scores for all conditions than were Hispanic and Other patients (eg, chest pain: Hispanic, 2.70 [95% CI, 2.69-2.71]; Other, 2.68 [95% CI, 2.65-2.70]; White, 2.59 [95% CI, 2.58-2.59]; both *P* < .001) compared with Black patients, except for shortness of breath, and compared with Asian patients for falls (Table 3). Applying an upper limit of age 50 years, differences in triage scores remained significant across all symptoms for Hispanic patients and across the same conditions for Black patients (eg, nausea/vomiting: Black, 2.98 [95% CI, 2.97-3.0]; White, 2.92 [95% CI, 2.90-2.93]; *P* < .001). Differences for patients identifying as Other remained significant for chest pain, nausea/vomiting, and abdominal pain.

**Table 3.** Emergency Severity Index and Visit Work Relative Value Unit Differences Between Racial and Ethnic Groups Stratified by Age Compared With White Patients

Chief symptom	American Indian or Alaska Native	Asian	Black	Hispanic	Native Hawaiian or Other Pacific Islander	Other^a^	White
**Emergency Severity Index score for patients age <65 y, mean (95% CI)**							
Abdominal pain	2.92 (2.85-2.99)	2.91 (2.89-2.92)	2.93 (2.92-2.94)	2.97 (2.96-2.97)	2.91 (2.80-3.01)	2.94 (2.92-2.95)	2.89 (2.89-2.89)
*P* value	.39	.03	<.001	<.001	.81	<.001	NA
Chest pain	2.64 (2.53-2.75)	2.63 (2.60-2.66)	2.65 (2.63-2.66)	2.70 (2.69-2.71)	2.54 (2.38-2.70)	2.68 (2.65-2.70)	2.59 (2.58-2.59)
*P* value	.38	.005	<.001	<.001	.57	<.001	NA
Shortness of breath	2.74 (2.59-2.88)	2.78 (2.74-2.83)	2.74 (2.71-2.76)	2.82 (2.80-2.83)	2.69 (2.43-2.95)	2.82 (2.78-2.85)	2.74 (2.73-2.75)
*P* value	.95	.06	.68	<.001	.75	<.001	NA
Nausea or vomiting	3.16 (2.99-3.33)	2.96 (2.91-3.01)	2.98 (2.97-3.0)	3.04 (3.02-3.05)	2.8 (2.54-3.06)	3.01 (2.98-3.04)	2.92 (2.90-2.93)
*P* value	.01	.08	<.001	<.001	.38	<.001	NA
Fall	3.2 (3.0-3.41)	3.2 (3.14-3.26)	3.21 (3.18-3.25)	3.24 (3.22-3.27)	3.5 (3.06-3.94)	3.18 (3.13-3.22)	3.09 (3.07-3.1)
*P* value	.27	<.001	<.001	<.001	.15	<.001	NA
**Emergency Severity Index score for patients age <50 y**							
Abdominal pain	2.95 (2.88-3.03)	2.92 (2.91-2.94)	2.94 (2.94-2.95)	2.98 (2.97-2.99)	2.9 (2.78-3.01)	2.95 (2.94-2.96)	2.91 (2.90-2.91)
*P* value	.24	.06	<.001	<.001	.88	<.001	NA
Chest pain	2.68 (2.55-2.82)	2.70 (2.67-2.74)	2.72 (2.7-2.73)	2.76 (2.75-2.77)	2.53 (2.36-2.69)	2.75 (2.72-2.78)	2.66 (2.65-2.67)
*P* value	.76	.03	<.001	<.001	.13	<.001	NA
Shortness of breath	2.69 (2.51-2.88)	2.81 (2.76-2.86)	2.78 (2.76-2.81)	2.84 (2.83-2.86)	2.73 (2.45-3.0)	2.85 (2.81-2.89)	2.79 (2.78-2.8)
*P* value	.30	.51	.46	<.001	.71	.004	NA
Nausea or vomiting	3.13 (2.96-3.31)	2.99 (2.94-3.04)	3.01 (2.99-3.03)	3.05 (3.04-3.07)	2.78 (2.49-3.07)	3.03 (2.99-3.06)	2.94 (2.93-2.95)
*P* value	.07	.08	<.001	<.001	.23	<.001	NA
Fall	3.17 (2.88-3.47)	3.28 (3.21-3.35)	3.28 (3.23-3.32)	3.29 (3.26-3.32)	3.5 (2.93-4.07)	3.23 (3.17-3.28)	3.18 (3.16-3.2)
*P* value	.97	.008	<.001	<.001	.37	.09	NA
**Work relative value units for patients age <65 y**							
Abdominal pain	4.01 (3.35-4.68)	3.92 (3.81-4.02)	4.44 (4.38-4.49)	4.19 (4.15-4.23)	4.49 (3.55-5.43)	3.96 (3.87-4.04)	3.92 (3.89-3.95)
*P* value	.73	.95	<.001	<.001	.25	.43	NA
Chest pain	3.36 (2.73-3.98)	3.52 (3.37-3.66)	4.28 (4.21-4.35)	4.10 (4.05-4.16)	3.85 (3.15-4.55)	3.95 (3.82-4.07)	3.38 (3.34-3.42)
*P* value	.94	.09	<.001	<.001	.34	<.001	NA
Shortness of breath	3.06 (2.45-3.66)	3.28 (3.07-3.48)	3.99 (3.9-4.09)	3.71 (3.63-3.79)	4.65 (2.82-6.49)	3.38 (3.21-3.54)	3.29 (3.24-3.35)
*P* value	.55	.90	<.001	<.001	.11	.34	NA
Nausea or vomiting	3.65 (2.58-4.72)	3.31 (3.05-3.58)	3.80 (3.68-3.91)	3.41 (3.33-3.49)	3.35 (2.51-4.20)	3.28 (3.08-3.47)	3.44 (3.37-3.51)
*P* value	.73	.38	<.001	.61	.92	.14	NA
Fall	3.54 (2.55-4.53)	3.48 (3.25-3.72)	4.05 (3.90-4.20)	3.90 (3.78-4.02)	5.18 (2.06-8.31)	3.72 (3.51-3.94)	3.95 (3.88-4.03)
*P* value	.53	.004	.33	.56	.42	.07	NA

Abbreviation: NA, not applicable.

^a^ Other race and ethnicity category is patient defined. It was available for any patient who did not feel that the predefined categories were appropriate.

In this sample, White patients’ visits were not associated with a greater number of total wRVUs compared with patients of other racial and ethnic backgrounds. Black patients’ visits were associated with more wRVUs for all reasons for presentation other than falls, compared with White patients (eg, chest pain: Black, 4.32 [95% CI, 4.25-4.39]; White, 3.55 [95% CI, 3.52-3.58]; *P* < .001) (Table 2). Hispanic patients’ visits were associated with more wRVUs for abdominal pain, chest pain, and shortness of breath, and patients identifying as Other were assigned more wRVUs per visit for chest pain (Other, 3.96 [95% CI 3.84-4.08]; White, 3.55 [95% CI, 3.52-3.58]; *P* < .001).

When controlling for less-acute triage scores, these differences were seen over a wider variety of chief symptoms. Black patients’ visits were associated with a greater number of wRVUs across all 5 reasons for visiting the ED (shortness of breath: Black, 3.76 [95% CI, 3.65-3.86]; White, 2.86 [95% CI, 2.80-2.91]; *P* < .001) (Table 4). Hispanic patients’ visits were associated with more wRVUs for all reasons for presentation other than falls, and patients identifying as Other were associated with more wRVUs per visit for both chest pain and abdominal pain (abdominal pain: Hispanic, 4.45 [95% CI, 4.4-4.51]; Other, 3.84 [95% CI, 3.75-3.94]; White, 3.62 [95% CI, 3.59-3.65]; *P* < .001).

**Table 4.** Visit Work Relative Value Unit Differences Between Racial and Ethnic Groups Stratified by Triage Emergency Severity Index Score Compared With White Patients

Chief symptom	American Indian or Alaska Native	Asian	Black	Hispanic	Native Hawaiian or Other Pacific Islander	Other^a^	White
**Work relative value units for patients with Emergency Severity Index score 3**							
Abdominal pain	3.8 (3.06-4.54)	3.81 (3.7-3.92)	4.45 (4.4-4.51)	4.2 (4.16-4.24)	4.56 (3.5-5.62)	3.84 (3.75-3.94)	3.62 (3.59-3.65)
*P* value	.48	.001	<.001	<.001	.05	<.001	NA
Chest pain	3.08 (2.33-3.83)	3.26 (3.08-3.44)	3.9 (3.82-3.98)	3.77 (3.71-3.83)	3.62 (2.56-4.68)	3.33 (3.18-3.47)	3.04 (3.0-3.08)
*P* value	.92	.02	<.001	<.001	.32	<.001	NA
Shortness of breath	2.71 (2.08-3.33)	3.22 (2.96-3.47)	3.76 (3.65-3.86)	3.58 (3.48-3.67)	3.97 (1.49-6.45)	3.08 (2.9-3.26)	2.86 (2.80-2.91)
*P* value	.73	.004	<.001	<.001	.20	.02	NA
Nausea or vomiting	2.98 (2.3-3.67)	3.23 (2.95-3.51)	3.82 (3.7-3.94)	3.53 (3.45-3.62)	3.09 (2.28-3.9)	3.36 (3.13-3.6)	3.19 (3.12-3.26)
*P* value	.73	.83	<.001	<.001	.91	.17	NA
Fall	3.64 (2.29-4.99)	4.14 (3.75-4.54)	4.78 (4.59-4.98)	4.40 (4.25-4.55)	4.17 (1.68-6.66)	4.52 (4.24-4.81)	4.12 (4.06-4.18)
*P* value	.52	.89	<.001	.003	.98	.004	NA

Abbreviation: NA, not applicable.

^a^ Other race and ethnicity category is patient defined. It was available for any patient who did not feel that the predefined categories were appropriate.

When controlling for patients aged 65 years or older, Black patients’ visits were associated with a greater number of wRVUs for all symptoms except falls, Hispanic patients’ visits were associated with a greater number of wRVUs for chest pain, abdominal pain, and shortness of breath, and visits by patients identifying as Other were associated with more wRVUs for chest pain (Black, 4.28 [95% CI, 4.21-4.35]; Hispanic, 4.10 [95% CI, 4.05-4.16]; Other, 3.95 [95% CI, 3.82-4.07]; White, 3.38 [95% CI, 3.34-3.42]; *P* < .001) (Table 3). These associations remained when controlling for patients aged 50 years and older.

Patients who did not speak English as a primary language were assigned less acute ESI scores for chest pain, abdominal pain, and nausea/vomiting (chest pain: primary non-English, 2.62 [95% CI, 2.60-2.63]; primary English, 2.59 [95% CI, 2.58-2.59]; *P* < .001). However, their visits were associated with higher wRVUs for all chief symptoms, except nausea/vomiting (eg, abdominal pain: primary non-English 4.24 [95% CI, 4.20-4.29]; primary English, 4.10 [95% CI, 4.08-4.12]; *P* < .001) (Table 5). Patients without an in-network primary care physician were also assigned less acute ESI scores for chest pain, shortness of breath, and falls (eg, falls: PCP out-of-network, 3.00 [95% CI, 2.98-3.01]; in-network PCP, 2.94 [95% CI, 2.93-2.95]; *P* < .001). Their visits were also associated with a small decrease in the number of associated wRVUs for chest pain only (PCP out-of-network, 3.71 [95% CI, 3.66-3.75]; in-network PCP, 3.82; [95% CI, 3.79-3.85]; *P* < .001).

**Table 5.** Emergency Severity Index and Visit Work Relative Value Units Differences Between Patients With English as Primary vs Non-Primary and Non-English Speakers and Those With vs Without In-Network Primary Care

Chief symptom	All	Primary language			PCP		
		English	Non-English	*P* value	In network	Out of network	*P* value
**Emergency Severity Index score, mean (95% CI)**							
Abdominal pain	2.91 (2.91-2.91)	2.9 (2.9-2.9)	2.96 (2.95-2.96)	<.001	2.91 (2.91-2.91)	2.92 (2.91-2.92)	.008
Chest pain	2.59 (2.59-2.6)	2.59 (2.58-2.59)	2.62 (2.60-2.63)	<.001	2.57 (2.56-2.57)	2.65 (2.64-2.66)	<.001
Shortness of breath	2.72 (2.71-2.73)	2.72 (2.71-2.72)	2.74 (2.72-2.76)	.02	2.70 (2.69-2.71)	2.77 (2.76-2.78)	<.001
Nausea or vomiting	2.94 (2.93-2.95)	2.93 (2.93-2.94)	3.0 (2.98-3.02)	<.001	2.94 (2.93-2.95)	2.95 (2.94-2.96)	.03
Fall	2.96 (2.95-2.97)	2.96 (2.95-2.96)	2.99 (2.97-3.01)	.006	2.94 (2.93-2.95)	3.0 (2.98-3.01)	<.001
**Work relative value units**							
Abdominal pain	4.12 (4.1-4.14)	4.10 (4.08-4.12)	4.24 (4.20-4.29)	<.001	4.10 (4.08-4.13)	4.16 (4.12-4.19)	.009
Chest pain	3.78 (3.76-3.81)	3.7 (3.67-3.73)	4.38 (4.31-4.45)	<.001	3.82 (3.79-3.85)	3.71 (3.66-3.75)	<.001
Shortness of breath	3.67 (3.64-3.71)	3.61 (3.57-3.65)	4.23 (4.11-4.34)	<.001	3.68 (3.64-3.73)	3.65 (3.58-3.71)	.33
Nausea or vomiting	3.62 (3.57-3.66)	3.6 (3.55-3.65)	3.79 (3.66-3.92)	.007	3.65 (3.6-3.71)	3.55 (3.48-3.63)	.04
Fall	4.52 (4.48-4.56)	4.44 (4.4-4.48)	5.19 (5.05-5.33)	<.001	4.5 (4.45-4.54)	4.57 (4.5-4.64)	.10

Abbreviation: PCP, primary care physician.

## Discussion

In this multicenter cross-sectional study of patients presenting to the ED, we found that White patients with the same chief symptoms were frequently assigned more acute ESI scores compared with Black patients, Hispanic patients, and patients identifying as having an Other racial or ethnic background. These findings are broadly consistent with those of Joseph et al,^4^ and the acute nature of these symptoms suggests that racial and ethnic disparities in triage cannot be explained primarily in terms of differences in visits for nonurgent care.^15^ These disparities likely have structural, institutional, and interpersonal causes.

White patients in our study tended to be older than patients in any other racial and ethnic group. This presents a potential confounder, as older patients are more likely to present with emergent causes for many of these chief symptoms, such as chest pain^16^ and abdominal pain,^17^ and are more likely to experience traumatic injuries from falls,^18^ which could justify assigning them more acute triage scores. However, this must be viewed in light of the fact that studies have identified a tendency toward undertriaging older ED patients to less acute ESI scores, suggesting that the age of the White population in our study was unlikely to explain the discrepancy.^5,19^ We found that these racial and ethnic discrepancies in triage ESI persisted for most conditions when stratifying for age, even with a conservative threshold.

The potential negative outcomes of these disparities in triage are manifold and can lead to immediate and downstream harms to patients. The most straightforward is that undertriaging patients with life-threatening symptoms directly endangers them—-patients with chest pain and active ischemia have demonstrably worse outcomes when physician evaluations, electrocardiograms, and interventions are delayed.^20,21^ In the context of this study, even small statistical differences between who is assigned an ESI level of 2 rather than 3 can have outsized clinical impacts, as patients assigned ESI 2 are considered unstable and prioritized for available beds. Amortized over tens of thousands of patients, even 0.1- to 0.2-point differences represent long delays in evaluation for many patients with acute symptoms, such as chest pain.

Patients who have more acute medical conditions are less tolerant of prolonged wait times and more likely to leave without being seen by a physician.^22^ Undertriaging patients with acute, but not immediately life-threatening symptoms leads to longer wait times for physician evaluations, entailing delays in time-sensitive treatments, such as antibiotics in patients with sepsis,^23^ and for imaging in patients with fractures.^24^ Lower acuity scores can also lead to a mismatch in the degree of care patients need and are given in the ED, as patients with lower triage ESI scores are more likely to be placed in areas with higher patient-to-nurse ratios.^25,26^

Racial and ethnic disparities in triage also cause immediate and delayed harms by undermining the trustworthiness of the ED and the health care system as a whole. Structural, institutional, and interpersonal racism, discrimination, and bias in health care—both historic and current—have undermined the trustworthiness of health care institutions for many communities primarily of minority racial and ethnic populations.^27,28,29^ The interplay between such disparities, mistrust of the health care system, and resulting limited engagement with preventive and acute care are associated with worse clinical outcomes.^30,31,32,33^ As it is often the first point of contact with the health care system, disparities in ED triage may further exacerbate these harms.

Our findings differ from previous studies considering the total number of wRVUs associated with patients’ ED visits, as we did not find a higher visit intensity for White patients.^4^ Instead, we found that patients with Black, Hispanic, and Other racial and ethnic backgrounds tended to have higher wRVUs associated with their visits for many symptoms. As White patients in our study tended to be older and might be expected to have higher visit acuity, these findings suggest that the racial and ethnic disparities observed in triage may be more consequential than they might initially appear.

Our secondary analysis suggests that patients’ primary language may be an underlying contributor to differences in triage ESI scores and overall workup intensity. Triage is often conducted under substantial time constraints, and the use of an interpreter can potentially consume much of the time available for the triage assessment; thus, nurses may feel pressured to use an ad hoc interpreter or limit the duration of the interview, missing important details and leading to undertriage.^34^ Conversely, when physicians face similar barriers to communication, they may compensate by ordering more diagnostic tests, such as advanced imaging, assuming that they are missing important details in their history-taking.^35,36^

Together, these findings suggest that there exist major disparities between the triage scores assigned to patients in different racial and ethnic groups and the depth of physician evaluations these patients ultimately receive. Clear measures to address these disparities are critical. While there is enthusiasm for providing implicit bias training to frontline clinical staff, there is limited evidence to demonstrate that this translates into concrete improvements in bias or outcomes.^37,38^

Structural institutional solutions are essential, in addition to training to address implicit bias, racism, and discrimination. One potential solution to facilitate debiasing triage is the use of machine learning and clinical decision support systems to help objectively assign triage scores.^12,39,40^ This approach has inherent appeal in that a number of systems have shown superior accuracy to traditional triage methods, and when used in conjunction with explainable models, can help clinicians feel involved in the decision-making process.^41,42^ However, it is essential that researchers developing such systems are aware of potential disparities in their data sets and develop the systems with an eye toward mitigating disparities,-or else they risk reinforcing, rather than alleviating, these same biases.^43^ At least one clinical decision support system implementation has been shown to reduce existing institutional disparities.^44^ These and other systems-based solutions may create more durable solutions to triage disparities.

### Limitations

This study has several limitations. Our study examines wRVUs as a surrogate for visit intensity but does not directly examine rates of advanced imaging or intravenous analgesia, which have been unequally distributed in prior studies.^8,9,10,11^ For patients without prior visits within our network, demographic information is often collected after triage and, as a result, can be incomplete. Our study also did not directly examine negative clinical outcomes resulting from undertriage, such as mortality, as such events are relatively rare. Approximately 6308 visits (2.5%) represent repeated presentations by patients with the same chief symptoms during the 4-year study period.

The grouping of patients into a Hispanic ethnic category elides substantial heterogeneity between racial, ethnic, national, and linguistic distinctions. Patients who chose primarily to identify as Black, but who also identify as of Hispanic ethnicity were not included within the Hispanic category. Likewise, patients who identify primarily as White or Other, but indicated Spanish as their primary language or nationality from a primarily Spanish-speaking country (eg, Guatemala) were not counted within the Hispanic category, potentially diluting associations.

## Conclusions

In this cross-sectional study across the EDs of a hospital network, we found that patients with Black, Hispanic, and Other racial and ethnic backgrounds were often assigned less-acute triage scores for the same chief symptoms compared with White patients, despite the fact that patients from these groups often had more involved ED visits. Patients’ primary language may be an important factor contributing to these disparities. Structural interventions to address institutional and interpersonal discrimination and bias, such as machine learning and clinical decision support algorithms, may provide a means of debiasing the triage process, but caution is needed to ensure that biased data do not filter into the data sets that train these algorithms.

## Notes

Supplement.
Data Sharing Statement
