# Supplementary material for: Race and Ethnicity and Primary Language in Emergency Department Triage
Source: JAMA Netw Open. 2023 Oct 12;6(10):e2337557. doi: 10.1001/jamanetworkopen.2023.37557 (PMC10570890; doi:10.1001/jamanetworkopen.2023.37557)
Supplement: Supplement. — Data Sharing Statement [file jamanetwopen-e2337557-s001.pdf]

# Data Sharing Statement

Joseph. Race and Ethnicity and Primary Language in Emergency Department Triage. *JAMA Netw Open*. Published October 12, 2023. doi:10.1001/jamanetworkopen.2023.37557

**Data**
**Data available:** No
